# Selenium protects against nesfatin‐1 modulation of the hypothalamic‐pituitary‐testicular axis during hypothyroidism in male rats

**DOI:** 10.14814/phy2.15923

**Published:** 2024-01-24

**Authors:** Rehab Ahmed Ahmed El‐Shaer, Sarah Ibrahim, Passant Medhat Hewady, Marwa Mohamed Atef, Omnia Safwat El‐Deeb, Yasser Mostafa Hafez, Rania Saed Amer, Jehan Abd El‐Hameed El‐Sharnoby, Norhan Ahmed AbuoHashish, Marwa Mahmoud Awad

**Affiliations:** ^1^ Medical Physiology Department, Faculty of Medicine Tanta University Tanta Egypt; ^2^ Human Anatomy and Embryology Department, Faculty of Medicine Tanta University Tanta Egypt; ^3^ Biochemistry Department, Faculty of Medicine Tanta University Tanta Egypt; ^4^ Internal Medicine Department, Faculty of Medicine Tanta University Tanta Egypt; ^5^ Clinical Pathology Department, Faculty of Medicine Tanta University Tanta Egypt; ^6^ Pharmacology Department, Faculty of Medicine Tanta University Tanta Egypt

**Keywords:** hypothalamic‐pituitary‐testicular axis, hypothyroidism, nesfatin‐1, propylthiouracil, selenium

## Abstract

Normal gonadal function can be disrupted by hypothyroidism. Hypothyroidism disturbs testicular function directly and centrally by affecting the hypothalamic‐pituitary‐testicular axis with unclear mechanism. As nesfatin‐1 neurons co‐localized with TRH and GnRH neurons in the hypothalamus, it could play a role in centrally hypothyroidism induced testicular dysfunction. Selenium (Se), by affecting thyroid iodide supply, could relieve these disturbances. So, we aim to identify the role of nesfatin‐1 as a link between testicular dysfunction and hypothyroidism through modulating the MAPK/ERK pathway while discussing the possible role of Se in alleviating hypothyroidism and associated testicular damage. Forty male rats were divided equally into: Control: distilled water, Se: Se orally, Propylthiouracil (PTU): PTU orally, PTU + Se: Se with PTU orally. Serum thyroid function, gonadal hormones, nesfatin‐1, testicular redox status, sperm analysis, brain tissue GnRH, nucleobindin 2‐derived polypeptide, pMAPK/ERK gene expression, histological changes and immunohistochemical expression of testicular proliferating cell antigen (PCNA) were done. PTU induced hypothyroidism and reduction of gonadal hormones which both were correlated with reduced nesfatin‐1. There was testicular stress with reduced GnRH, NUCB2, pMAPK/ERK gene expression, and PCNA immunopositive cells. These parameters were reversed by Se. Nesfatin‐1 could be the central link between hypothyroidism and disturbances of the hypothalamic pituitary testicular axis.

## Graphical abstract

An overview diagram highlighting the role of nesfatin‐1 as a link between testicular dysfunction induced by propylthiouracil hypothyroidism through modulating the MAPK/ERK pathway with the possible role of Se in alleviating hypothyroidism and associated testicular damage.

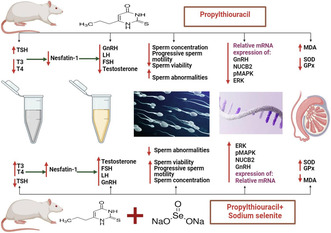

## 1 INTRODUCTION

The testis, a bean‐shaped male reproductive gland, measuring three to five centimeters in length and two to three centimeters in width, plays a dual role as both endocrine and exocrine glands (Tsili et al., 2019). It produces sperm and androgens under the control of the anterior pituitary gland, which releases luteinizing hormone (LH) that regulates testosterone levels and follicle‐stimulating hormone (FSH) that regulates sperm production (Tiwana & Leslie, 2023).

The complementary function of spermatogenic cells, supporting cells, and interstitial cells for generation, differentiation, and maturation of sperm is mainly governed by the hypothalamus‐pituitary‐gonadal axis (Kaftanovskaya et al., 2012).

One of the pivotal hormones necessary for the processes of spermatogenesis and steroidogenesis is the thyroid hormone (T3) that impacts the growth and maturation of testicles (La Vignera & Vita, 2018). Another well‐known physiological impact of thyroid hormones is altering the generation of reactive oxygen species (ROS)–induced by the cellular oxidative stress pathway (Naseem et al., 2019).

Research has proven that a lack of thyroid hormone can trigger not only hypothyroidism but also oxidative stress, and regression of fertility and testicular function (Kamel & Hamouli‐Said, 2018). In men, hypothyroidism is associated with hypogonadism (Krysiak et al., 2020). Furthermore, hypothyroidism can affect the morphology and motility of sperm (Krassas & Markou, 2019).

In men, hypothyroidism can lead to hypogonadotropic hypogonadism due to an impact on the pituitary‐testicular axis, as it diminishes the levels of gonadotropin and testosterone hormones (Donnelly et al., 2013). Despite this, there have been limited studies exploring the mechanism of pituitary‐testicular axis imbalance present (Gołyński et al., 2019).

The 82‐amino acid protein (Nesfatin‐1) is derived from the 396‐amino acid precursor protein nucleobindin 2 (NUCB2). Nesfatin‐1 is a newly discovered anorexigenic peptide found in the hypothalamic paraventricular nucleus (PVN), supraoptic nucleus, arcuate nucleus, and lateral hypothalamic area and among other brain areas involved in food and metabolic regulation (Ayada et al., 2015). Moreover, the pituitary gland, cerebrospinal fluid, and brain stem all contain nesfatin‐1 (Prinz & Stengel, 2016).

Nesfatin‐1 was first discovered to have anorexigenic ability; however, additional research revealed that it also has cardiovascular effects, lipid metabolism, reproductive activities, and emotion‐related functions (Gao et al., 2016; Prinz & Stengel, 2016).

Nesfatin‐1 has been identified to be expressed in a variety of tissues, including reproductive organs (Dore et al., 2017). Several studies have found nesfatin‐1 expression in the male reproductive systems of humans and rats (Garcia‐Galiano et al., 2010; Kim et al., 2011) suggesting that nesfatin‐1, as a local regulator, controls the function of Leydig cells and is important in testicular function (Kim & Yang, 2012).

The regulation of energy balance and reproduction is intricately linked through neuroendocrine pathways. Among several hormones that impact food intake and energy balance, nesfatin‐1 is believed to have a significant role in this regulation, alongside resistin, adiponectin, growth hormone‐releasing hormone, and orexin. These hormones may also play a part in the gonad and contribute to the central regulation of reproduction (Gao et al., 2016).

The MAPK/ERK signaling pathway (mitogen‐activated protein kinase/extracellular signal‐regulated kinase signaling pathway) plays an important role in the transmission of cell signals from the cytoplasm to the nucleus through transduction systems that includes ligands, transmembrane receptors, and cytoplasmic secondary messengers, where they influence gene expression that regulates important cellular processes such as cell growth, proliferation, and apoptosis (Brzezianska & Pastuszak‐Lewandoska, 2011).

Rupp et al. (2021), reported that central nesfatin‐1 injection increased MAPK activity, ultimately increasing ERK1/2 phosphorylation in PVN neurons.

Selenium (Se) is an essential trace element that, due to its antioxidant characteristics, plays critical roles in immune function, thyroid hormone metabolism, and human reproductive health (Ramírez‐Acosta et al., 2022). Se plays a critical role in gonadal development, gametogenesis, and fertilization, likely by triggering antioxidant defense mechanisms and pathways that are sensitive to changes in redox conditions (Graupner et al., 2015).

Se supplements enable the conversion of reactive oxygen species (ROS) into less reactive molecules through seleno‐protein synthesis. These impact redox‐regulated genes and promote a healthy balance in the body (Hugejiletu et al., 2013). Also, Se is essential for spermatogenesis in spermatozoa (Qazi et al., 2019). Previous research has linked Se deficiency to a variety of reproductive problems, including aberrant testicular morphology, decreased sperm quality, and altered sperm structure and fertilization capacity (Ahsan et al., 2014; Varlamova, 2016).

Considering the information presented above, our objective was to establish the possibility that nesfatin‐1 could serve as a potential link between disruptions in the hypothalamic‐pituitary‐testicular axis (HPT) and the development of hypothyroidism induced by propylthiouracil in a male rat model. Additionally, we sought to investigate the potential protective effect of Se consumption in these rats.

## 2 MATERIALS AND METHODS

### 2.1 Chemicals

#### 2.1.1 Sodium selenite

The white powder of sodium selenite (Na_2_SeO_3_) was purchased from Sigma Aldrich's product number (229865‐5GM). 1 mL of distilled water was used to dissolve 10 μg of sodium selenite to be given in a dose of 10 μg/kg that was chosen according to a previous study (Lamfon, 2014).

#### 2.1.2 Propylthiouracil (PTU)

It was purchased in the form of Thyrocil® white discoid tablets (Amoun Pharmaceutical Co., Egypt), each one contains 50 mg of the active ingredient. Each tablet was dissolved in 8.3 mL of distilled water, generating 1 mL containing 6 mg of PTU, to be administered at the 6 mg/kg dosage as stated by (Villar et al., 1998).

### 2.2 Animal care

The animals utilized in this study were 40 male albino rats of the local strain, aged 16–18 weeks and weighing 180–220 g. In a standard laboratory environment (12‐h light/dark cycles, an ambient temperature of 20–25°C, and full access to food and drink), The animals were kept in clean, well‐ventilated cages with five rats per cage. Before starting the experimental procedure, the rats were acclimatized for 2 weeks. To compensate for the number of deaths in rats, 20% were added to each group as dropouts.

### 2.3 Ethics statement

This study was evaluated and authorized by Ethical Committee of Medical Research, Faculty of Medicine, Tanta University, Egypt (Approval code: 36264PR123/2/23) that follows the National Institutes of Health Guide for the Care and Use of Laboratory Animals (NIH Publication No. 8023, revised 1978).

### 2.4 Experimental design

Animals were randomly assigned into four groups of 10 rats each:

**Control group** (10) rats: 1 mL of distilled water was given orally daily for six weeks.

**Selenium group (Se) group** (10) rats: Rats were given sodium selenite in a dose similar to that of the PTU + Se group for 6 weeks (Lamfon, 2014).

**Propylthiouracil (PTU) group** (10) rats: These animals received PTU in a dose (6 mg/kg) which was given by oral gavage at 6 mg/mL distilled water daily for 6 weeks (Villar et al., 1998).

**Propylthiouracil (PTU)** + **selenium (Se) group** (10) rats: Rats were given sodium selenite in a dose (10 μg/kg) which was given by oral gavage at 10 μg/mL distilled water daily for 6 weeks with PTU administration that was given at the same dose and duration as the PTU group (Lamfon, 2014).

### 2.5 Blood sampling

At the end of the experimental procedure, intraperitoneal 0.1 mL of 1% sodium barbiturate (Novelli et al., 2007) was the anesthesia that was given to the overnight starved animals. A heart puncture was used to collect blood samples, and serum was obtained after centrifuging the samples for 3000 cycles per minute. Then, in a freezer at −80°C, the divided, extracted serum aliquots were kept for later use.

### 2.6 Tissue collection and tissue homogenate preparation

After blood sampling, rats were sacrificed by decapitation then, an anterior abdominal wall midline incision was made to excise both testes, and then they were weighed. Then saline rinsing to remove any blood contamination, then drying by blotting with filter paper. The right testis was preserved in 10% phosphate‐buffered formalin for histopathological and immunohistochemical evaluation, while the left testis was immediately frozen at −80°C for biochemical and molecular research.

The brain was extracted by decapitation and immediately frozen at −80°C for biochemical and molecular analysis.

Homogenization of the left testis and brain tissue was performed by five volumes of 50 mM phosphate buffer (pH 7.4), followed by 12,000 xg centrifugation at 4°C for 10 min was carried out for the homogenate using Centurion Scientific centrifuge (Model: K241, Serial No: 13783‐6, voltage: 230, Frequency: 50–60 hz Fuse, Type: T6‐3A). Storage in a freezer at −80°C until the time of analysis.

Finally, the sacrificed animals were then placed in special packaging in accordance with safety precautions and infection control measures.

### 2.7 Biochemical assays

#### 2.7.1 The following parameters were assessed

##### Thyroid function test

Using the ELISA technique, serum‐free T3, free T4, and TSH were measured by kits supplied by (MyBioSource, Inc., San Diego, USA) Cat Numbers. (MBS261285, MBS580037, and MBS2019160), respectively following the manufacturer's instruction.

##### Measurement of serum Nesfatin‐1

Using the ELISA technique, serum nesfatin‐1 was measured by kits supplied by (MyBioSource, Inc., San Diego, USA) Cat. Number. (MBS705284) following the manufacturer's instruction.

##### Assessment of hypothalamic pituitary testicular (HPT) axis

Using the ELISA technique, serum levels of (Gonadotropin‐releasing hormone) (GnRH), (Luteinizing Hormone) (LH), (Follicle‐Stimulating Hormone) (FSH) and (testosterone) were measured by kits supplied by (MyBioSource, Inc., San Diego, USA) Cat. Numbers. (MBS635755, MBS4500760, MBS2021901, and MBS4502799) respectively, following the manufacturer's instruction.

##### Assessment of parameters for redox status

Using the colorimetric method, testicular tissue Malondialdehyde (MDA), superoxide dismutase (SOD), and Glutathione peroxidase (GPX) levels were assessed by kits purchased from (Biodiagnostic, Dokki, Giza, Egypt) Cat. Numbers. (MD 2529, SD 2521, and GP 2524), respectively, following the manufacturer's protocol.

##### Reproductive assessment (evaluation of sperm parameters)

The epididymal cauda was cut with a scalpel blade in a Petri dish pre‐heated to 37°C. Then, 2 mL of saline was added to the caudal epididymal fluid for dilution (Narayana et al., 2002). One drop of well‐mixed sample was placed on specific slide, and the motility/concentration module of the computer‐assisted semen analysis (CASA) system was performed using Mira Lab‐Egypt (Mira 9000 sperm Analyzer CASA software). Sperm quality is determined by characteristics such as sperm concentration, motility, and viability (Emadi et al., 2012). Sperm viability and morphologic abnormalities can be determined by staining with eosin‐nigrosine dye. Then, under a light microscope with a magnification of 1000, the red‐stained spermatozoa are dead, while the white‐stained ones are alive (Ezzatabadipour et al., 2012). Sperm count, motility, and total sperm abnormality were determined using an Olympus trinocular microscope (Mohamed & Abdelrahman, 2019).

#### 2.7.2 RNA extraction and quantitative RT‐PCR detection of nucleobindin 2‐derived polypeptide (NUCB2), GnRH, and phosphorylated mitogen‐activated protein kinase/extracellular signal‐regulated kinase (pMAPK/ERK) in the brain tissue

##### RNA extraction

To isolate total RNA from the brain tissue, The PureLink® RNA Mini Kit (Life Technologies Corporation, USA) was used according to the manufacturer's instructions (Thermo Scientific, USA, # k 0731). Total RNA concentration and purity were measured using a NanoDrop spectrophotometer (NanoDrop Technologies, Inc., Wilmington) at the OD260 and OD260/280 ratios, respectively, and then stored at −80°C.

##### Complementary DNA (cDNA) synthesis

A 5 μg total RNA sample was used for cDNA synthesis using Reverse Transcriptase (a revert Aid H Minus) (Thermo Scientific, USA, # Ep0451) following the manufacturer's instructions.

NUCB2, GnRH, and pMAPK/ERK relative gene abundance was evaluated using the newly acquired cDNA (as a template) via the Step One Plus Real‐Time PCR equipment (Applied Biosystem). Primer 5.0 software was used to design the primer sequences; the primers for the above‐mentioned genes are demonstrated in (Table 1).

**TABLE 1:** Sequence of primers for the PCR reaction.

Gene name	Primer sequence	GenBank accession no.
NUCB2	FOR: 5′TTTGAACACCTGAACCACCA3′ REV: 5′ TGGTCTTCGTGCTTCCTCTT3′	MK_804767
GnRH	FOR: 5′ TCAGCTGCCTGTTCATCATC 3′ REV: 5′AACATTTCCGGATCAAACCA3′	NM_031038
Rat p38 MAPK	FOR: 5′TTGGAGGTAACCAGGAGGGT3′ REV: 5′GTATAGACGGCAATTGGGGG3′	NM_001164043.1
Rat ERK	FOR: 5′‐CTCTGTCATTGCCACCA‐3′ REV: 5′‐ATTCCACTCTCCATCTCCAT‐3′	NM_002745.5
Rat GAPDH	FOR: 5′ CAACTCCCTCAAGATTGTCAGCAA 3′ REV: 5′ GGCATGGACTGTGGTCATGA 3′	NM_001394060.2

Abbreviations: FOR, forward primer, REV, reverse primer.

The conditions of the thermal cycler are as follows. After a 10‐min denaturation at 95°C, 40–45 amplification cycles were performed (DNA denaturation for 15 s at 95°C, annealing for 30 s at 60°C, and extension for 30 s at 72°C). The temperature was raised from 63 to 95°C at the end of the previous cycle for melting curve analysis. Ct values (cycle thresholds) were calculated for both (target and housekeeping: GAPDH) genes and the relative gene abundance assessment was carried out utilizing the (Livak & Schmittgen, 2001) 2−∆∆Ct technique of analysis as follows:

Relative expression = 2 −ΔΔCt, where ΔCt = (CT of target gene–CT GAPDH), and this was calculated for the target group and control ΔΔCt = (ΔCt of the target group‐ ΔCt of control) (Livak & Schmittgen, 2001).

### 2.8 Histological and immunohistochemical assessment of testicular tissue

#### 2.8.1 Histological examination of the testicular tissues

Paraffin blocks were constructed by embedding formalin‐fixed right testicular tissue in paraffin wax samples, and routine histological analysis of 5–6 μm thickness was done by staining with hematoxylin and eosin (Jackson, 2013). An Olympus light microscope model BX43F was used for examination. Using image analysis tools (ImageJ, 1.46a, NIH, Bethesda, MD, USA), a morphometric study was conducted using 10 non‐overlapping readings obtained for each slide in all groups, and the mean values were obtained.

#### 2.8.2 Histological morphometric analysis

Assessment of testicular damage and the process of spermatogenesis was performed histopathologically according to Johnsen's testicular biopsy score (Johnsen, 1970).

For histology assessment, from each animal, 10 tubules were graded at ×100 magnification, and each tubule was assigned a score from 1 to 10 based on the presence or absence of various types of cells in the seminiferous tubules, such as (spermatozoa, spermatids, spermatocytes, spermatogonia, germ cells, and Sertoli cells). A higher Johnsen score indicates greater spermatogenesis, while a lower one indicates a worse condition. A score of 10 indicates that there was complete spermatogenesis with many spermatozoa and germinal epithelium organized in regular thickness leaving an open lumen. A score of 9 indicates Many spermatozoa present but germinal epithelium disorganized with marked sloughing or obliteration of lumen, whereas a score of 8 indicates that only a few spermatozoa present in the section. A score of 7 indicates that no spermatozoa present but many spermatids are present and a score of 6 demonstrates that no spermatozoa and only a few spermatids are present. A score of 5 demonstrates that no spermatozoa, no spermatids but several or many spermatocytes present and of 4 indicates that only few spermatocytes and no spermatids or spermatozoa present, whereas a score of 3 indicates that spermatogonia are the only germ cells present, of 2 and 1 indicates that no germ cells but Sertoli cells present and no cells in the tubular section respectively.

### 2.9 Immunohistochemical analysis of the testicular tissue

Using immunohistochemical methods, in testicular tissue, the expression of proliferating cell nuclear antigen (PCNA) was evaluated. 1/5000 diluted IgG Monoclonal antibodies against PCNA (MyBioSource, Inc., San Diego, USA) Cat number (MBS6249233) were used for the incubation of dewaxed, rehydrated paraffin‐embedded sections. After that, the slides were incubated with a secondary biotin‐linked Goat Anti‐Rat IgG (H + L chain specific, secondary antibody, MyBioSource, Inc., San Diego, USA) Cat number (MBS674712) and with the streptavidin‐peroxidase complex. Sections were then incubated with the developing solution (diaminobenzidine‐hydrogen peroxide; DAKO), and counterstained with hematoxylin. The immune reaction was visualized as a brown reaction and counterstained with hematoxylin.

Tools for image analysis were used for morphometric studies, and the immunohistochemical image quantification was evaluated by (ImageJ, 1.46a, NIH, Bethesda, MD, USA) (Buchwalow & Böcker, 2010). The mean number of positive (PCNA) immunoreactive cells was counted in 10 non‐overlapping areas of each section at 400 magnifications.

### 2.10 Statistical analysis

The results were recorded as (mean ± SD). The Shapiro–Wilk test for normality was done before the start of analysis for all data. Results of Johnsen scores were analyzed using Kruskal–Wallis test followed by Dunn's multiple comparison test. For the rest of the data a one‐way analysis of variance (ANOVA) was used, followed by a post hoc test (Tukey). Moreover, Pearson's correlation (*r*) was used to examine the relationships between serum levels of nesfatin‐1 and the other parameters tested. *r* = (−1 to +1). Statistical analysis was done by using SPSS software (IBM SPSS Statistics for Windows, IBM Corp., Version 23.0, Armonk, NY, USA). Statistical significance was defined as *p* values less than 0.05, 0.01, or 0.001.

## 3 RESULTS

### 3.1 Effect of PTU and Se on thyroid function tests and serum nesfatin‐1

It was evidenced that PTU administration resulted in hypothyroidism. This was detected in our result as, when compared to the control group, there was a significant rise in serum TSH level with a significant decline in serum‐free T3, T4 in the PTU group. This state was associated with a concomitant significant decline in serum nesfatin‐1 level. While co‐administration of Se resulted in the correction of such a condition, it was evidenced by a significant decline in serum TSH level with a significant rise in serum‐free T3, T4, and a concomitant significant rise in serum nesfatin‐1 level in the PTU + Se group when compared to the PTU group. In addition, in the Se group, there was no significant change of the previously mentioned parameters when compared to the control group. (Table 2).

**TABLE 2:** Effect of PTU and Se on thyroid function tests and serum nesfatin‐1 among all studied groups.

Parameter/group	Control group, *n* = 10	Se group, *n* = 10	PTU group, *n* = 10	PTU+ Se group, *n* = 10	F ratio	*p* value
Serum TSH (μIU/mL)	3.56 ± 1.03	3.42 ± 1.23	15.08 ± 1.94^a^ ^,^ ^b^	4.12 ± 1.30^c^	161.99	0.000
Serum free T3 (ng/mL)	3.90 ± 1.46	5.26 ± 1.34	2.20 ± 0.95^a^ ^,^ ^b^	4.02 ± 1.40^c^	9.300	0.000
Serum free T4 (μg/dL)	5.32 ± 1.11	4.88 ± 1.75	1.95 ± 0.62^a^ ^,^ ^b^	5.06 ± 1.20^c^	16.41	0.000
Serum nesfatin‐1 (ng/mL)	4.47 ± 1.86	4.99 ± 1.68	2.30 ± 0.95^a^ ^,^ ^b^	4.30 ± 1.65^c^	5.629	0.003

*Note*: Data are presented as mean ± SD. One‐way ANOVA test was used for statistical comparisons, followed by the (Tukey) post hoc test. Superscripts a, b and c denote a statistically significant difference at *p* < 0.05.

Abbreviations: *n*, number of experimental rats; TSH, thyroid‐stimulating hormone; T3, triiodothyronine; T4, thyroxine.

^a^ *p* < 0.05 in comparison with control group.

^b^ *p* < 0.05 in comparison with Se group.

^c^ *p* < 0.05 in comparison with PTU group.

**FIGURE 1:**
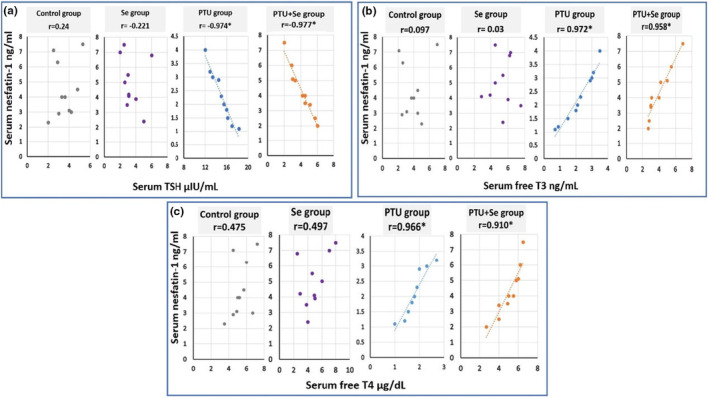
Correlation of (a) serum TSH (μIU/mL), (b) free T3 (ng/mL), and (c) free T4 (μg/dL) with serum nesfatine‐1 (ng/mL) in all studied groups. Number of rats: 10. Superscript *denotes statistical significance at *p* < 0.05. The Pearson correlation coefficient (Pearson *r* test) was utilized to determine the strength and relationship between two variables. *r* = (−1 to +1). A value of −1 indicates a strong negative correlation. A value of +1 indicates a strong positive correlation. A value of 0 indicates that there is no association. TSH stands for (Thyroid‐stimulating hormone) T3 stands for (Triiodothyronine) T4 stands for (Thyroxine).

Moreover, there was a significant negative correlation between serum TSH level and serum nesfatin‐1 level, with a significant positive correlation between serum free T3, T4, and serum nesfatin‐1 level in both the PTU group and the PTU + Se group (Figure 1).

### 3.2 Effect of PTU and Se on the hypothalamic‐pituitary‐testicular (HPT) axis

To investigate the effect of hypothyroidism on the HPT axis, serum (GnRH, LH, FSH, and testosterone) levels were detected. Their levels showed a significant decline following PTU administration in comparison to the control group. While co‐administration of Se resulted in a significant rise in their levels as compared to the PTU group. However, Se alone had no significant impact on HPT when compared to the control group (Table 3).

**TABLE 3:** Effect of PTU and Se on hypothalamic‐pituitary‐testicular axis among all studied groups.

Parameter/group	Control group, *n* = 10	Se group, *n* = 10	PTU group, *n* = 10	PTU+ Se group, *n* = 10	F ratio	*p* value
Serum GnRH (ng/mL)	5.00 ± 1.70	5.54 ± 1.65	2.16 ± 0.72^a^ ^,^ ^b^	4.79 ± 1.60^c^	10.454	0.000
Serum LH (mIU/mL)	4.31 ± 1.71	5.30 ± 1.63	1.92 ± 0.63^a^ ^,^ ^b^	4.69 ± 1.77^c^	9.651	0.000
Serum FSH (mIU/mL)	2.12 ± 1.12	3.85 ± 1.31	1.10 ± 0.48^a^ ^,^ ^b^	2.09 ± 0.79^c^	13.602	0.000
Serum testosterone (ng/mL)	5.01 ± 1.46	6.02 ± 1.76	1.60 ± 0.43^a^ ^,^ ^b^	5.61 ± 1.74^c^	19.308	0.000

*Note*: The mean ± standard deviation are used to express the data. The one‐way ANOVA test was used for statistical comparisons, followed by the (Tukey) post hoc test. Superscript a, b and c denote a statistically significant difference *p* < 0.05.

Abbreviations: FSH, Follicle stimulating hormone; GnRH, Gonadotropin‐Releasing Hormone; LH Luteinizing Hormone; *n*: the number of experimental rats.

^a^ *p* < 0.05 in comparison with control group.

^b^ *p* < 0.05 in comparison with Se group.

^c^ *p* < 0.05 in comparison with PTU group.

**FIGURE 2:**
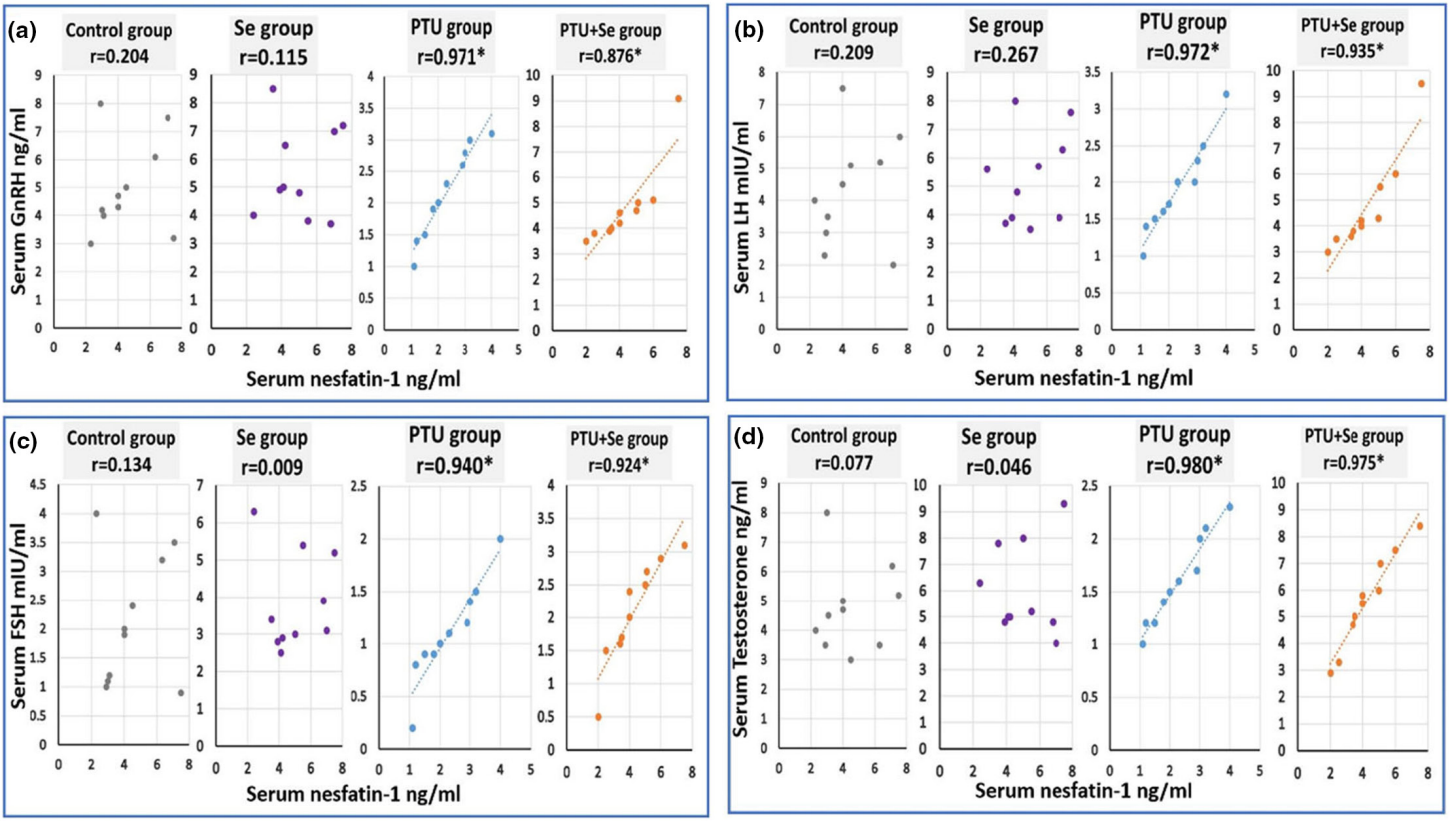
Correlation of (a) serum GnRH (ng/mL), (b) LH (mIU/mL), (c) FSH (mIU/mL), and (d) testosterone (ng/mL) with serum nesfatine‐1 (ng/mL) in all studied groups. Number of rats: 10. Superscript * denotes statistical significance at *p* < 0.05. The Pearson correlation coefficient (Pearson *r* test) was utilized to determine the strength and relationship between two variables. *r* = (−1 to +1). A value of −1 indicates a strong negative correlation. A value of +1 indicates a strong positive correlation. A value of 0 indicates that there is no association. GnRH stands for (Gonadotropin‐Releasing Hormone) LH stands for (Luteinizing Hormone) FSH stands for (Follicle stimulating hormone).

Moreover, there was a significant positive correlation between serum GnRH, LH, FSH, and testosterone levels and serum nesfatin‐1 levels in both the PTU group and the PTU + Se group (Figure 2).

### 3.3 Effect of PTU and Se on redox status in testicular tissue

The oxidative stress state was evaluated by measuring MDA, SOD, and GPx levels in testicular tissue. Hypothyroidism‐induced by PTU administration resulted in a significant rise in testicular MDA level with a significant decline in testicular SOD and GPx levels as compared to the control group, indicating a state of oxidative stress. Meanwhile, co‐administration of Se resulted in a significant decline in testicular MDA with a significant rise in testicular SOD and GPx levels as compared to the PTU group. So, Se probably has antioxidant activity. Moreover, Se alone had significantly increased GPx when compared to control group, but it had no significant impact on SOD, and MDA when compared with the control group as shown in (Table 4).

**TABLE 4:** Effect of PTU and Se on redox status in testicular tissue among all studied groups.

Parameter/group	Control group, *n* = 10	Se group, *n* = 10	PTU group, *n* = 10	PTU+ Se group, *n* = 10	F ratio	*p* value
Testicular MDA level (nmol/g tissue)	39.63 ± 6.88	35.31 ± 7.91	63.34 ± 9.10^a^ ^,^ ^b^	40.15 ± 6.46^c^	27.40	0.000
Testicular SOD level (U/mg tissue protein)	18.58 ± 3.20	19.08 ± 3.81	6.88 ± 1.34^a^ ^,^ ^b^	18.05 ± 3.24^c^	36.98	0.000
Testicular GPx level (U/mg tissue protein)	7.54 ± 1.56	9.71 ± 2.31^a^	3.34 ± 1.16^a^ ^,^ ^b^	8.36 ± 1.74^c^	24.87	0.000

*Note*: The mean ± standard deviation are used to express the data. The one‐way ANOVA test was used for statistical comparisons, followed by the (Tukey) post hoc test. Superscript a, b and c denote a statistically significant difference *p* < 0.05.

Abbreviations: GPx, Glutathione peroxidase; MDA, Malondialdehyde; *n*, the number of experimental rats; SOD, Superoxide dismutase.

^a^ *p* < 0.05 in comparison with control group.

^b^ *p* < 0.05 in comparison with Se group.

^c^ *p* < 0.05 in comparison with PTU group.

### 3.4 Effect of PTU and Se on relative mRNA gene expression of nucleobindin 2‐derived polypeptide (NUCB2), GnRH, and pMAPK/ERK in brain tissue

**FIGURE 3:**
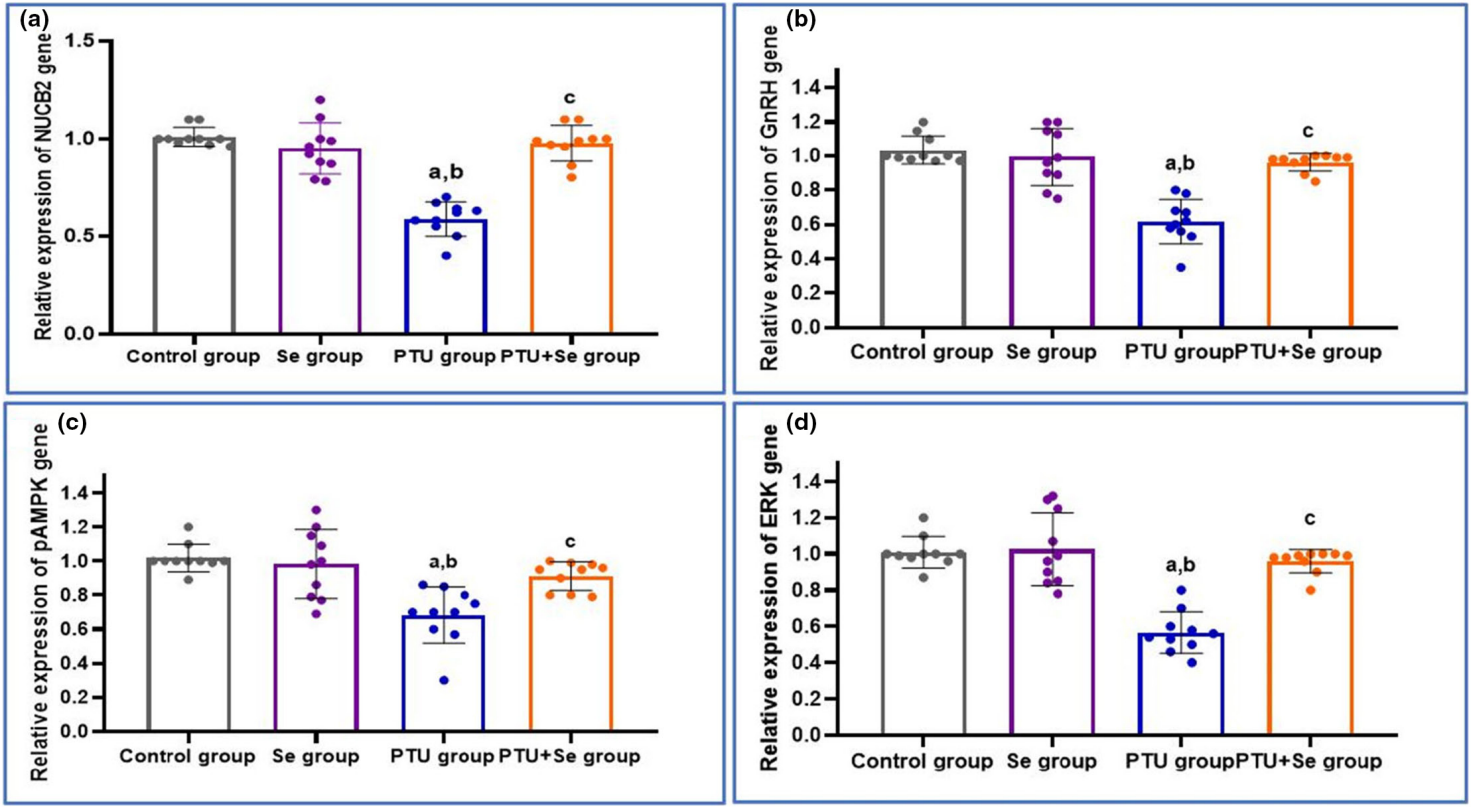
Effect of PTU and Se on relative mRNA gene expression of NUCB2, GnRH, and pMAPK/ERK in brain tissue among all studied groups. Ten rats in each group. The mean ± standard deviation and individual data are used to express the data. The one‐way ANOVA test was used for statistical comparisons, followed by the (Tukey) post hoc test. Superscript a, b, and c denote a statistically significant difference at *p* < 0.05. ^a^ *p* < 0.05 in comparison of control group. ^b^ *p* < 0.05 in comparison of Se group. ^c^ *p* < 0.05 in comparison of PTU group. NUCB2 stands for (Nucleobindin 2‐derived polypeptide) GnRH stands for (Gonadotropin‐releasing hormone) pMPAK stands for (phosphorylated Mitogen‐activated protein kinase) ERK stands for (Extracellular signal‐regulated kinase).

In brain tissue, the relative mRNA gene expressions of NUCB2, GnRH, and pMAPK/ERK showed a significant downregulation following PTU administration when compared to the control group. While co‐administration of Se results in a significant upregulation of their expression when compared to the PTU group. This postulated a central effect of hypothyroidism in brain tissue. In addition, in the Se group, there was no significant change in the mRNA expression of the previously measured genes when compared with the control one (Figure 3).

### 3.5 Effect of PTU and Se on reproductive assessment

Testicular dysfunction can be defined as when the testicles cannot produce sperm. This was evidenced in the PTU group in the form of a significant decline in total sperm count, motility, and viability percentages that are associated with a significant rise in the % of sperm abnormalities when compared to the control group, while co‐administration of Se resulted in a significant correction of such conditions when compared to the PTU group. However, Se alone had no significant impact on sperm analysis when compared with the control group. (Table 5).

**TABLE 5:** Effect of PTU and Se on functional sperm parameters among all studied groups.

Parameter/group	Control group, *n* = 10	Se group, *n* = 10	PTU group, *n* = 10	PTU+ Se group, *n* = 10	F ratio	*p* value
Sperm count (million/mL)	86.90 ± 7.12	83.20 ± 7.96	23.60 ± 5.71^a^ ^,^ ^b^	81.70 ± 9.67^c^	152.26	0.000
Progressive sperm motility (% of total sperm counts)	67.00 ± 8.28	69.34 ± 12.04	30.50 ± 5.46^a^ ^,^ ^b^	64.70 ± 9.27^c^	40.91	0.000
Sperm viability (%)	93.50 ± 1.71	94.60 ± 1.71	74.90 ± 7.14^a^ ^,^ ^b^	92.80 ± 1.93^c^	58.27	0.000
Sperm abnormalities (%)	6.50 ± 1.71	5.40 ± 1.71	25.10 ± 7.14^a^ ^,^ ^b^	7.20 ± 1.93^c^	58.27	0.000

*Note*: The mean ± standard deviation are used to express the data. The one‐way ANOVA test was used for statistical comparisons, followed by the (Tukey) post hoc test. Superscript a, b and c denote a statistically significant difference at *p* < 0.05.

Abbreviations: *n*, the number of experimental rats.

^a^ *p* < 0.05 in comparison with control group.

^b^ *p* < 0.05 in comparison with Se group.

^c^ *p* < 0.05 in comparison with PTU group.

### 3.6 Effect of PTU and Se on histological and immunohistochemical assessment of testicular tissue

#### 3.6.1 Histological results (hematoxylin and eosin (H&E) staining)

**FIGURE 4:**
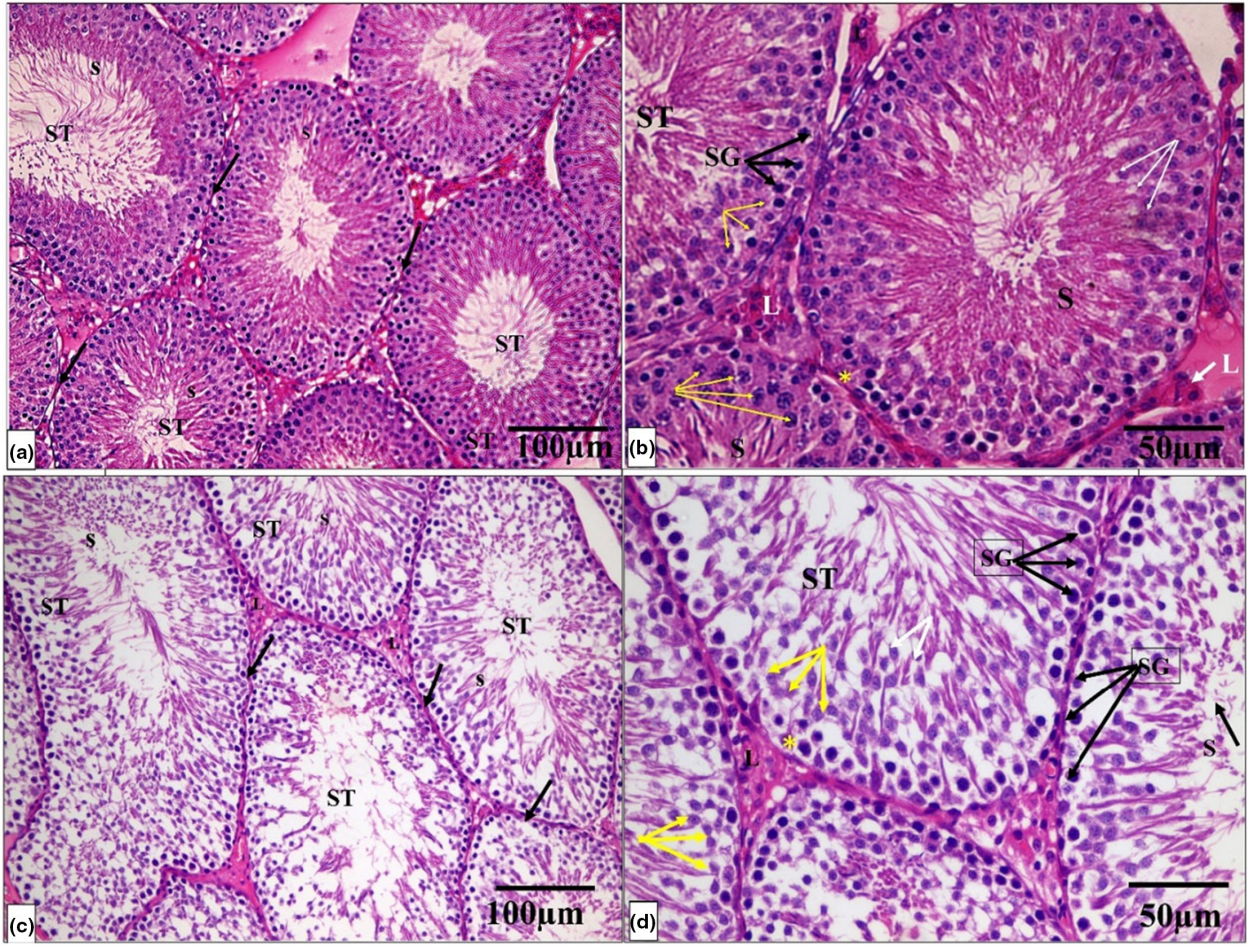
A photomicrograph of testicular tissue from the control and Se groups (a, c) show normal seminiferous tubules (ST) lined by germinal epithelium and Sertoli cells (*) resting on the basement membrane. Sperm (S) are seen in the lumen. Each tubule is surrounded by an intact basement membrane (thin black arrow). There is a normal amount of interstitial space in between the tubules, which contain Leydig cells (L). (b, d) A higher magnification photomicrograph showing the spermatogenic cells lining each tubule in the form of spermatogonia (black arrows SG), primary spermatocytes (yellow arrows), spermatids (white arrows), and sperms on the lumen (H & E × 200, scale bar = 100 μm and H & E × 400, scale bar = 50 μm).

The histological architecture of the control and Se groups' testicular tissue was normal. Many tightly packed, organized seminiferous tubules lined by germinal epithelium formed the testicular tissue. Each tubule is surrounded by a well‐defined basement membrane. Between the tubules, there were very small interstitial spaces occupied by connective tissue and clusters of Leydig cells. Numerous layers of spermatogenic cells, including spermatogonia, primary and secondary spermatocytes, spermatids, and sperm, formed the tubular germinal epithelium. Spermatogonia are tiny, spherical cells found in the tubules' basal region. Primary spermatocyte nuclei are larger and rounder than spermatogonia nuclei. Spermatids were identified as small, round cells with pale nuclei. Sperm with long tails have also been discovered in the lumen of tubules. Sertoli cells were pyramidal with big vesicular nuclei that appeared on the basement membrane (Figure 4a–d).

**FIGURE 5:**
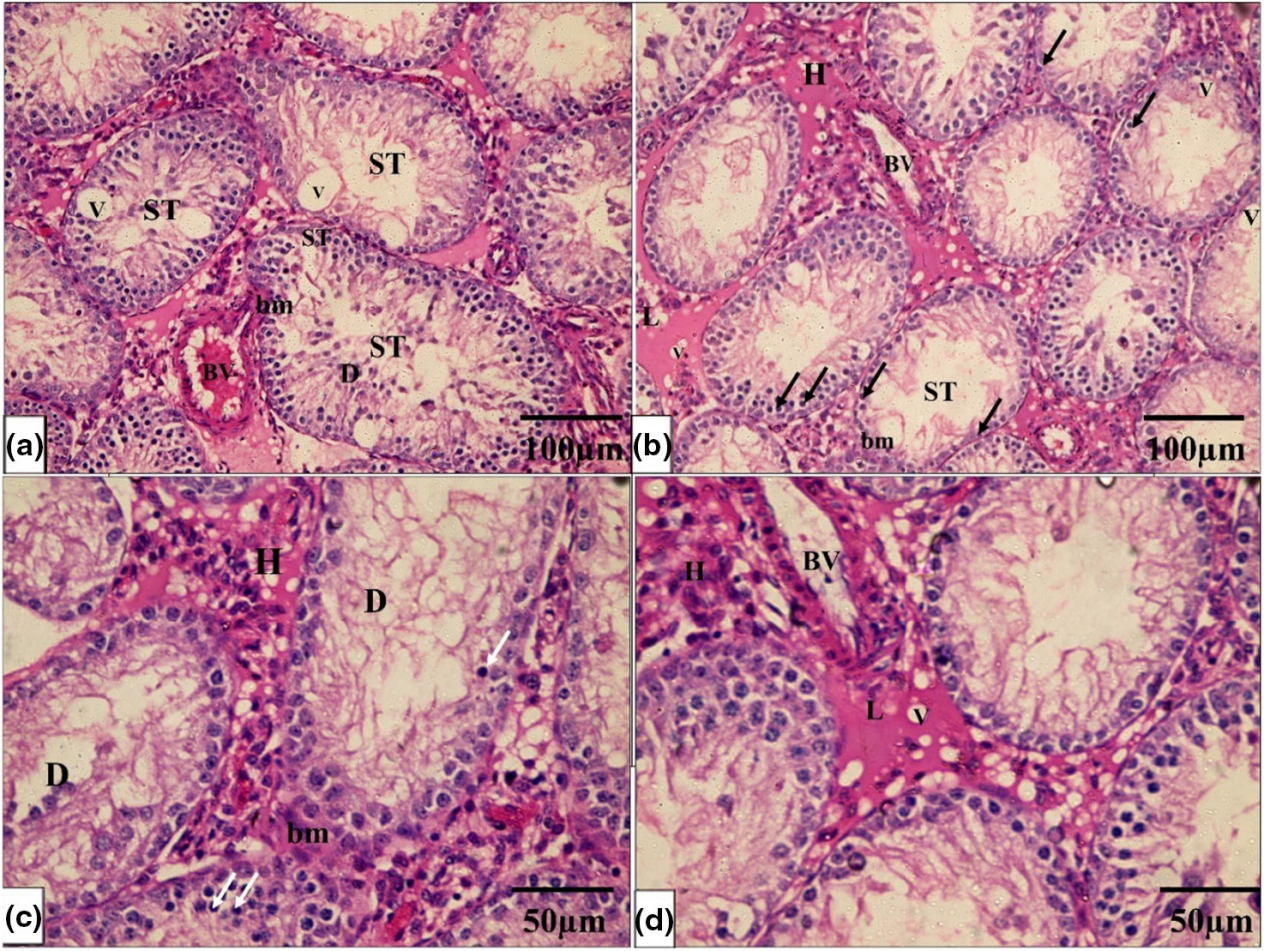
A photomicrograph of testicular tissue from the PTU group (a–d) shows seminiferous tubules (ST) with a noticeable decrease in the number of spermatogenic cells, disorganization, and vacuolation (V) of most germinal epithelial cells. Most of the tubule‐lining spermatogenic cells had small, dark pyknotic nuclei (double arrows). Desquamated and detached cells (D) are seen in some tubules' lumen. There are few Leydig cells (L) and vacuoles (V) in interstitial space tissue. The basement membrane (BM) surrounding the tubules is distorted and detached in some areas. Moreover, there are congested blood vessels (BV) in the interstitial tissue. (H & E × 200, scale bar = 100 μm and H & E × 400, scale bar = 50 μm).

While the PTU‐treated group showed that seminiferous tubules are disordered, with a significant decrease in the quantity, disarray, and vacuolation of most germinal epithelial cells, there was a lot of interstitial tissue between the tubules, but there were just a few Leydig cells and vacuoles. A hemorrhage was also discovered in the interstitial tissue. The majority of the tubules were lined by cells with darkly pigmented pyknotic nuclei and large gaps between them. Desquamated cells were discovered in several tubule lumens. There were also clogged blood vessels and detached sections of the basement membrane (Figure 5a–d).

**FIGURE 6:**
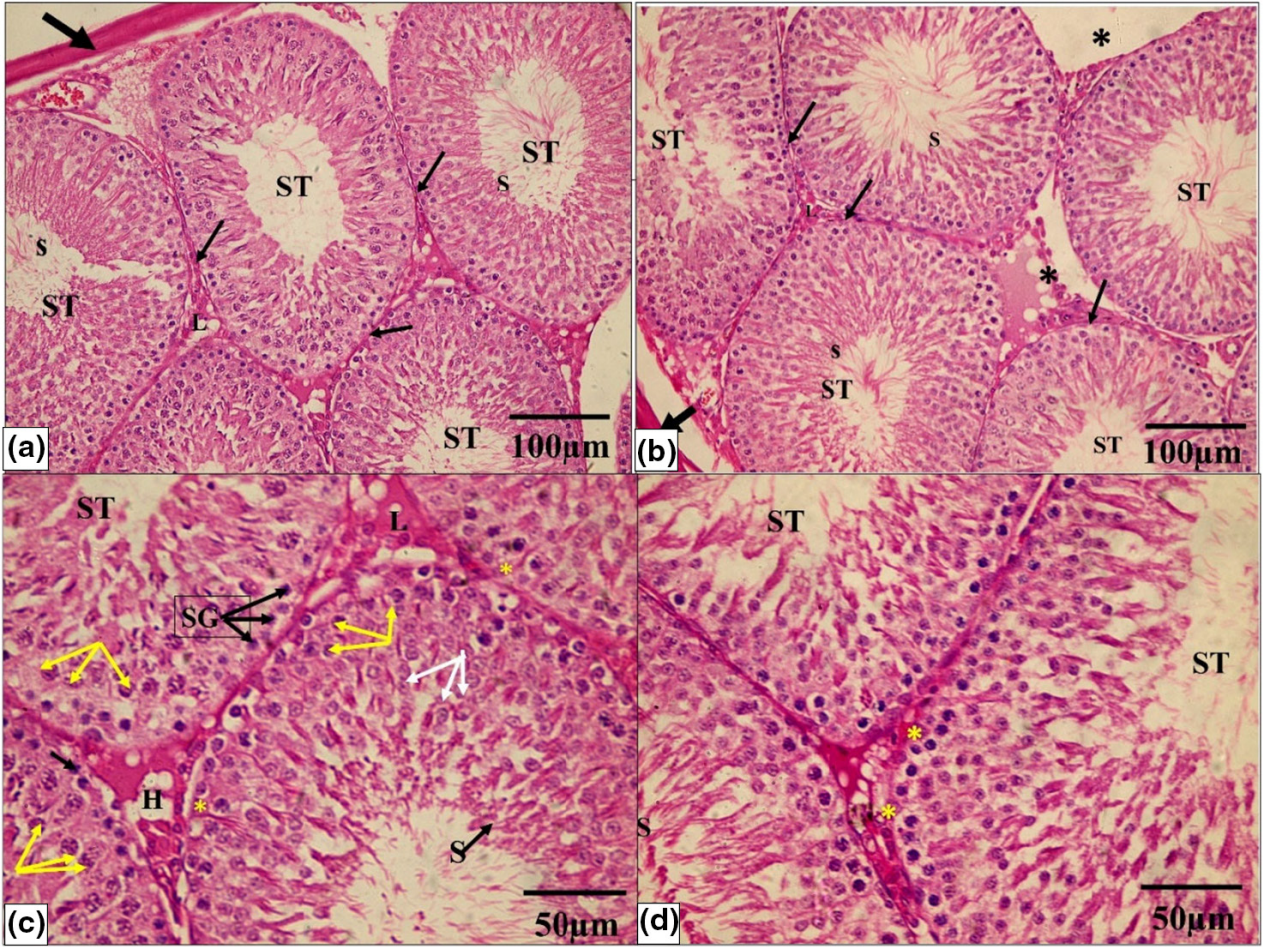
A photomicrograph of testicular tissue from the PTU+ Se group (a, b) shows normal seminiferous tubules (ST) lined by germinal epithelium. The tubules are surrounded by a thick fibrous capsule, tunica albuginea (thick black arrow). Sperm (S) are seen in the lumen. Each tubule is surrounded by an intact basement membrane (thin black arrow). There is a normal amount of interstitial space in between the tubules, which contain Leydig cells (L). Some tubules show wide interstitial tissue in between (*). (c, d) A higher magnification photomicrograph showing the spermatogenic cells lining each tubule in the form of spermatogonia (black arrows SG), primary spermatocytes (yellow arrows), spermatids (white arrows), and sperms on the lumen and Sertoli cells (yellow *) resting on the basement membrane. (H & E × 200, scale bar = 100 μm and H & E × 400, scale bar = 50 μm).

On the other hand, the PTU + Se group indicated that the seminiferous tubules had a normal histological structure. A basement membrane enveloped the tubules, which were lined by Sertoli cells and stratified germinal epithelium. Sperm was discovered in the tubule lumen. There was abundant interstitial tissue with hemorrhage between some seminiferous tubules (Figure 6a–d).

**FIGURE 7:**
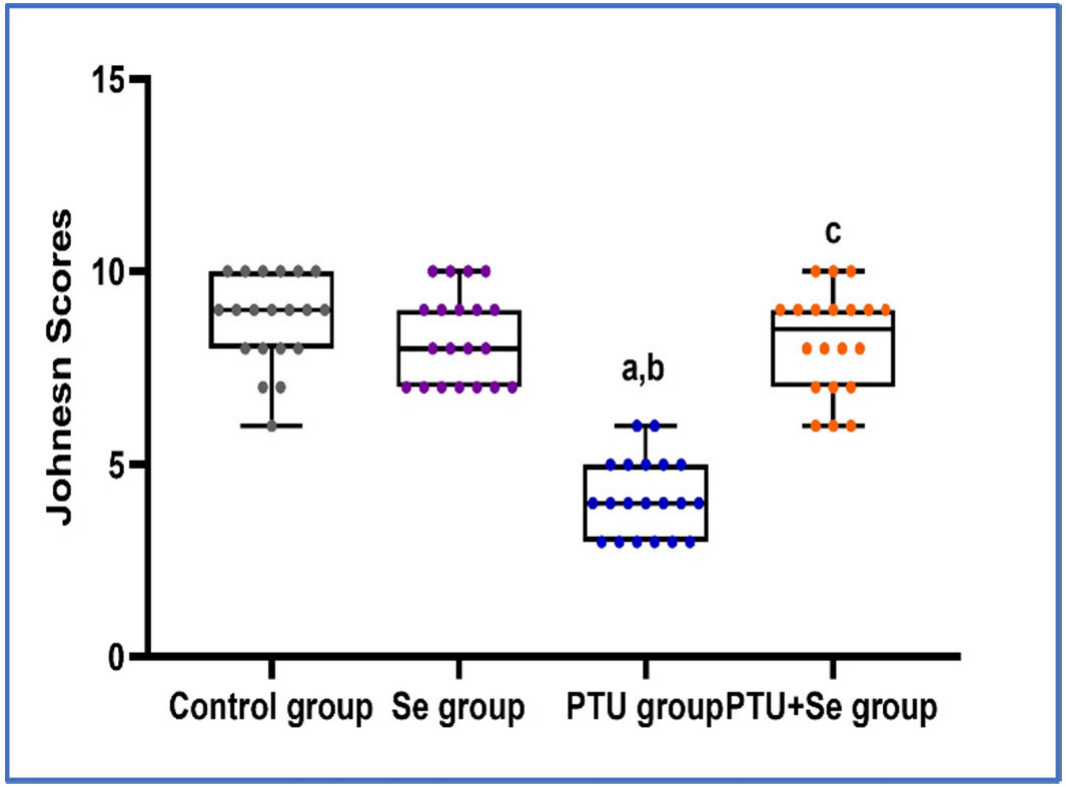
Effect of PTU and Se on the Johnsen scores of seminiferous tubular cross‐sections among all studied groups. Box plot was used to express the data. The bottom of the plot represents 25%, the middle represents the median and the top represent the 75% of the data. Superscript a, b, and c denote a statistically significant difference at (*p* < 0.05). ^a^ *p* < 0.05 in comparison of control group. ^b^ *p* < 0.05 in comparison of Se group. ^c^ *p* < 0.05 in comparison of PTU group by using Kruskal–Wallis test followed with Dunn's pairwise comparison post hoc test.

In all groups, the frequency distribution of Johnsen scores of seminiferous tubular cross‐sections was performed. It was found that scores 9–10 had the highest distribution in both the control and PTU + Se groups, with a relative frequency of 70% in the control group compared to 50% in the PTU cotreated with the Se group. In the PTU group, scores 3–4 had the highest prevalence, with a relative frequency of 65% (Table 6). With further statistical analysis, the mean rank of Johnsen score in the control group was 55.13, in the Se group was 48.48 while the mean rank of the PTU group was 10.70 with a statistically significant difference (*p* = 0.000) when compared to both groups. Whereas in the PTU + Se group, the mean rank was 47.70, which showed no significant difference (*p* = 0.306, 0.915) with the control group and the Se group respectively, with significant difference (*p* = 0.000) when compared to PTU group (Figure 7).

**TABLE 6:** Effect of PTU and Se on the frequency distribution of histopathological score according to Johnsen scores of seminiferous tubular cross‐sections in all studied groups.

Score	Control group		Se group		PTU group		PTU + Se group	
	Absolute frequency	Relative frequency, %	Absolute frequency	Relative frequency, %	Absolute frequency	Relative frequency %	Absolute frequency	Relative frequency, %
10	6	30	6	30	0	0	3	15
9	8	40	6	30	0	0	7	35
8	3	15	3	15	0	0	4	20
7	2	10	3	15	0	0	4	20
6	1	5	1	5	2	10	2	10
5	0	0	0	0	5	25	0	0
4	0	0	0	0	7	35	0	0
3	0	0	1	5	6	30	0	0
2	0	0	0	0	0	0	0	0
1	0	0	0	0	0	0	0	0
Total	20	100%	20	100%	20	100%	20	100%

### 3.7 Effect of PTU and Se on immunohistochemical results of testicular tissue

**FIGURE 8:**
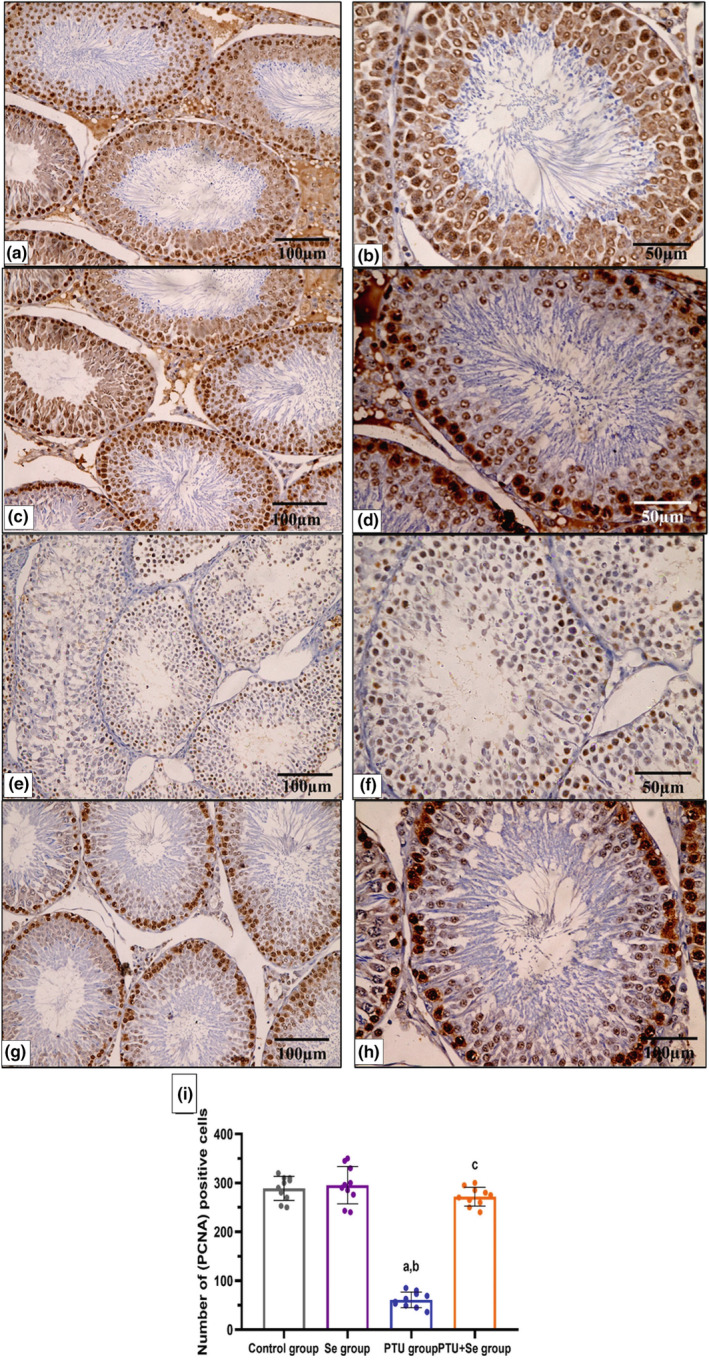
A representative photomicrograph of testicular tissue stained with PCNA immunostaining from the different groups. (a–d) Control and Se groups exhibit many positive PCNA immunoreactions in the form of brownish discoloration of the nuclei of germ cells. (e, f) The number of positive immune cells decreased in the PTU group. (g, h) PTU + Se group shows a moderate number of positive cells (PCNA‐immunostaining × 400, scale bar = 50 μm). (i) Number of (PCNA) positive cells, the mean ± standard deviation and individual data are used to express the data. The one‐way ANOVA test was used for statistical comparisons, followed by the (Tukey) post hoc test. Superscript a, b and c denote a statistically significant difference at (*p* < 0.05). ^a^ *p* < 0.05 in comparison of control group. ^b^ *p* < 0.05 in comparison of Se group. ^c^ *p* < 0.05 in comparison of PTU group.

Many PCNA‐immunopositive germ cells were seen in the seminiferous tubules of the control and Se groups (Figure 8a–d), while the PCNA‐immunopositive cells in the seminiferous tubules were few in the PTU group (Figure 8e,f). On the other hand, the PTU+ Se group had considerably more PCNA‐immunopositive cells in the seminiferous tubules (Figure 8g,h). A morphometric analysis of the number of PCNA‐immunopositive cells validated these findings. There was a significant decline in the number of immunopositive cells in the PTU group in comparison to the control group. While the PTU + Se group showed a significant elevation in the number of immunopositive cells in comparison to the PTU group (Figure 8i).

## 4 DISCUSSION

Primary hypothyroidism causes health issues in various physiological systems, affecting the hypothalamus anterior‐pituitary axis, leading to abnormalities in growth hormone, adrenocorticotrophic hormone, prolactin, and gonadotropin production (Donnelly et al., 2013). Testicular dysfunction due to hypothyroidism has been reported in various studies (Donnelly & White, 2000; Honbo et al., 1978). However, testicular function could be affected by micronutrient as Se. Se plays a crucial role in the reproductive system's control. According to nutritional research, Se is necessary for male fertility (Behne et al., 1996; Wu et al., 1973).

Nucleobindin 1 and NUCB2 are variants of the nucleobindin precursor. Through enzymatic action, NUCB2 generates nesfatin‐1, 2, and 3 (Barnikol‐Watanabe et al., 1994; Miura et al., 1992). Nesfatinergic neurons are abundant throughout the central nervous system and potentially regulate the hypothalamic‐pituitary‐gonadal axis (Oh‐I et al., 2006). Nesfatin‐1 is expressed in PVN and co‐localizes with neurons of thyrotropin‐releasing hormone (TRH), affecting their membrane potential (Brailoiu et al., 2007). Also, Nesfatin‐1's binding sites have been detected in the testis, pituitary, cortex, and hypothalamus's PVN (Prinz et al., 2016). Because nesfatin‐1 crosses the blood–brain barrier, it potentially influences GnRH neurons (Pan et al., 2007). In addition, nesfatin‐1 is located in GnRH neurons; hence, locally produced nesfatin‐1 may influence GnRH and other brain and pituitary‐derived reproductive hormones, and vice versa (Hatef & Unniappan, 2017).

Based on the previous finding the aim of this study was to identify testicular dysfunction resulting from hypothyroidism, through its central impact on the HPT axis and its peripheral effect on the testis, with investigating the underlying causes of HPT axis disturbances in hypothyroidism. This is the first study that investigates the role of nesfatin‐1 as regulator of the HPT axis through the MAPK/ERK signaling pathway. Additionally, the research explored the protective role of Se against hypothyroidism.

In the current study, hypothyroidism caused by PTU was indicated by a decrease in serum‐free T3 and free T4 and an increase in serum TSH through negative feedback. This was also observed in previous investigations (Sahoo et al., 2008). PTU's anti‐thyroid impact is achieved by the reduction of thyroid hormone production and inhibition of the transition of T4 to T3 (Alkalby & Alzerjawi, 2013). On the other hand, in the PTU+ Se group, serum‐free T3 and free T4 levels were significantly increased, and the TSH level was significantly decreased, this is attributed to the higher concentration of Se in thyroid gland, and it is nature as a part of seleno‐proteins, which are key component in thyroid hormone synthesis and metabolism (Schomburg, 2012). Se also participates in the conversion of T4 to T3 as the iodothyronine 5′‐deiodinase is a seleno‐enzyme (Mao et al., 2016).

As regards the central impact of primary hypothyroidism, previous studies suggest that hypothyroidism can impact the hypothalamic–pituitary axis, causing hypogonadotropic hypogonadism through decrement of GnRH levels and lowering of gonadotropin production (Krassas et al., 2010; Toni et al., 2005). Moreover, Oh‐I S et al. (Chandrasekhar et al., 1985), reported decrease in LH content of hypothyroid rats, which impair the LH response to GnRH. Additionally, hypothyroidism lowers sex hormone‐binding globulin levels, resulting in fewer sex hormones in the bloodstream (Selva & Hammond, 2009). In addition, T4 replacement enhances free testosterone levels that are usually reduced in hypothyroidism (Donnelly & White, 2000). These previous findings support the outcomes of this study as the data revealed a significantly low serum level of GnRH, LH, FSH, and testosterone in the PTU group, which also came in line with previous studies (Alkalby & Alzerjawi, 2013; El‐Kashlan et al., 2015). In contrast, no detectable changes in the level of these hormones were reported by other studies (Donnelly & White, 2000; Velazquez & Arata, 1997). This controversy may be caused by the difference in duration of treatment and/or the hypothyroidism induction method in experimental animals. The crossover between the hypothalamic‐pituitary‐thyroid and hypothalamic‐pituitary‐gonadal axes is not yet fully recognized, Hatef et al. (Tohei, 2004), postulated that hypothyroidism can directly affect the sensitivity of gonadotrophs to GnRH and can interfere with the production of LH, FSH, and testosterone.

It is postulated that the reduced testosterone will have a negative feedback effect on the HPT axis with subsequent elevation of LH, FSH, and GnRH, but from the results of the present work, it is evident that in the PTU group, hypothyroidism has a central effect on the HPT axis due to the reduction of GnRH relative mRNA expression in brain tissue with subsequent reduced serum GnRH, LH, FSH, and testosterone, thus creating a hypo‐gonadotrophic state. These results are in accordance with Velazquez and, Arata (Schomburg, 2012).

To determine the underlying central impact of hypothyroidism on HPT axis, we measured nesfatin‐1. It was reduced in the PTU group, and there was a negative correlation between TSH and nesfatin‐1 and a positive correlation between T3 and T4 and nesfatin‐1. This indicates that hypothyroidism is associated with a decrease in nesfatin‐1. This agreed with Liu et al. (2014). The negative correlation between TSH and nesfatin‐1 and the positive correlation with T3 and T4 were also reported by previous studies (Gungunes et al., 2014; Tohma et al., 2015). Also, in the PTU group, there was a positive correlation between serum nesfatin‐1 and serum GnRH, LH, FSH, and testosterone. These correlations suggest that nesfatin‐1 could be a potential link between hypothyroidism and disturbance of the HPT axis.

The impact of nesfatin‐1 on GnRH is controversial, Guvenc et al. (2019), found that nesfatin‐1 intracerebroventricular injection has no effect on altering GnRH plasma levels in male rats. However, in our study, we hypothesized that in PTU hypothyroid rats, reduction of relative mRNA NUCB2 expression and subsequent reduction of serum nesfatin‐1 are triggers of the reduced relative mRNA expression of GnRH with subsequent hypogonadotropic state. This agreed with Hatef & Unniappan (2017), who stated that in the hypothalamus, nesfatin‐1 directly stimulates GnRH mRNA and protein expression, suggesting that it might imitate kisspeptin in stimulating GnRH secretion. Furthermore, within pituitary gonadotropes, nesfatin‐1 acts on LβT2 cells to stimulate LHβ, through the up‐regulation of GnRH receptors. Also, it was reported that rats treated intracerebroventricularly with nesfatin‐1 had a remarkable increase in plasma FSH, LH, and testosterone levels (Guvenc et al., 2019). However, other studies hypothesized that nesfatin‐1 suppresses the expression of GnRH mRNAs (Pan et al., 2007; Price TO et al., 2007; Rajeswari & Unniappan, 2020). This conflicting effect of nesfatin‐1 on relative mRNA GnRH expression could be attributed to GnRH pulsatile release.

Concerning the cellular mechanism of action of nesfatin‐1, the results of the present study, revealed a significant reduction in brain tissue relative mRNA expression of pMAPK/ERK in the PTU hypothyroid group, and this could be explained by reduced brain relative mRNA expression of NUCB2 and nesfatin‐1, which is a stimulator for this pathway. In consistence with this, Tanida et al. (2015), reported that central nesfatin‐1 administration increased MAPK activity and ERK phosphorylation in PVN corticotropin‐releasing neurons. Moreover, the simulative effect of nesfatin‐1 on synapsin I through corticotropin‐releasing hormone receptor‐1 is mediated via the cAMP/MAPK/ERK pathway in human neuroblastoma cells (Chen et al., 2018). In addition, the impact of nesfatin‐1 on MAPK/ERK was proved by other studies, which found that MAPK/ ERK is the pathway that is activated by nesfatin‐1, but 5′ AMP‐activated protein kinase (AMPK) and phosphatidylinositol‐3‐kinases (PI3K) are not involved (Angelone et al., 2013; Ishida et al., 2012; Tanida et al., 2015). However, this disagrees with Tan et al. (2016), who postulated that nesfatin‐1 reduced the expression of ERK1/2, p38 MAPK, and mTOR in smooth muscle cells of the human gastrointestinal tract.

Because ERK proteins (the key components of MAPK signaling pathways) play a pivotal role in controlling the transcriptional response of pituitary cells' gonadotropin α subunit to GnRH (Kanasaki et al., 2012), and the suppression of the MAPK/ERK pathway leads to diminished sensitivity and the reaction of gonadotrophs to GnRH (Tohei, 2004). Therefore, an inhibition of the pMAPK/ERK pathway by the reduced nesfatin‐1 in the PTU group could be the cause of reduced (LH, FSH, and testosterone). On the other hand, the previous recorded hypogonadotropic state was prevented in the PTU+ Se group, this could be due to the ability of Se to prevent hypothyroidism which was further confirmed by the negative correlation between TSH and nesfatin‐1, and the positive correlation of T3, and T4 with nesfatin‐1 in the PTU + Se group. The heightened serum nesfatin‐1 levels coupled with upregulation of pMAPK/ERK signaling, enhances the responsiveness of gonadotrophs to GnRH. Notably, a positive correlation between nesfatin‐1 and serum (GnRH, LH, FSH, and testosterone) persists in the PTU+ Se treated group. In addition, as the present study showed that Se alone had a nonsignificant impact on the HPT axis, we hypothesized that Se by mitigation of the hypothyroid condition caused by PTU and consequently increasing nesfatin‐1 levels, might alleviate the negative central impact of hypothyroidism on the HPT axis.

In terms of the local effect of hypothyroidism on testicular function, thyroid hormones are believed to be one of the key elements in the control of cell metabolism and hence play a crucial role in oxidative stress (Petrulea et al., 2012). The findings of this study revealed that in PTU‐induced hypothyroidism, antioxidants were inhibited with an increase in lipid peroxidation, reducing testicular enzymatic defenses via a significant decline in antioxidants which was demonstrated by an increase in testicular MDA and a decrease in testicular GPX and SOD. This is in agreement with Selva and, Hammond (Sahoo et al., 2008). However, there are conflicting findings regarding oxidative stress in hypothyroidism; while a decrease in ROS production was expected due to hypothyroidism's hypometabolic state, other studies found no significant changes in lipid peroxidation or even an increase in the oxidative stress response (Chakrabarti et al., 2016; Messarah et al., 2007). The deficiency of iodine in hypothyroidism induces the inhibition of cellular antioxidants with a redox imbalance (Nanda et al., 2008). Also, high TSH directly triggers the oxidative stress response (Bhanja & Chainy, 2010). Moreover, hypothyroidism enhances ROS production through dysfunction of the respiratory chain in the mitochondria (Resch et al., 2002).

It was recognized that spermatogenesis and steroidogenesis in Leydig cells are highly susceptible to oxidative stress due to the accelerated cell division rate of these cells and the high consumption of oxygen by mitochondria in testicular tissue (Asadi et al., 2017). So, oxidative stress recorded in the PTU group can directly inhibit Leydig cells' testosterone secretion by suppressing the activity of several steroidogenic enzymes (Chen et al., 2008), as well as a decrease in testosterone synthesis by lowering the mitochondrial membrane potential required for testosterone synthesis (Allen et al., 2006), in addition to blocking both the expression and activation of proteins that regulate steroidogenesis (Diemer et al., 2003), all of which collectively decreased testosterone production. However, in the PTU+ Se group, the oxidative stress was prevented. This is in line with Swathy et al. (2006). This could be explained by Se's antioxidant impact and its capacity to boost glutathione levels in tissue, allowing it to restore antioxidant defenses that reduce hazardous amounts of hydrogen peroxide and lipid hydroperoxides. Se is involved in the production of many seleno‐proteins that are important for maintaining redox equilibrium (Owumi et al., 2020). GPX is a selenium‐dependent enzyme, so it increases in both Se and the PTU + Se groups (De Vega et al., 2016). GPX, in turn, inhibits lipid peroxidation and reduces MDA (Carmo de Carvalho e Martins et al., 2022).

In the PTU group, the decrease in sperm count, motility, and viability, as well as a significant increase in sperm abnormality indicates impact of hypothyroidism on spermatogenesis; this finding was consistent with previous studies (Sahoo et al., 2008; Wang et al., 2015). These findings were attributed to the drop in thyroid hormone which is a crucial mediator for germ cell differentiation and maturation (Singh et al., 2011), together with the decrement of the sex hormones (Capel, 2000; Kerr et al., 2006), in addition to oxidative stress that impacts sperm cells' motility and genetic integrity (Iommiello et al., 2015). However, in the PTU+ Se group, there was no impairment of spermatogenesis, due to the normal testosterone levels and the antioxidant impact of Se on Leydig cells, which improves their responsiveness to LH, this was in harmony with Owumi et al. (2020).

The effect of PTU‐induced hypothyroidism was confirmed by histological changes of the testis, which showed disorganized seminiferous tubules with vacuolation of most germinal epithelial cells and wide interstitial tissue between the tubules with hemorrhage in the interstitial, these changes are in alignment with (Algaidi et al., 2022).

The immunohistochemical expression of PCNA confirmed these changes. PCNA is required for DNA replication and repair in eukaryotic cells and declines as cells become inactive (Miura et al., 2002). In this work, PCNA‐positive cells in the testis of hypothyroid rats in the PTU group were significantly reduced, indicating DNA damage and that the actively dividing part of the germinal epithelium is damaged with a lack of repair capacity. This corresponded to the findings of (Fadlalla et al., 2017). Moreover, in PTU+ Se group, there was normal testicular architecture together with an increase in the number of immunopositive PCNA cells, which was in harmony with previous study (Owumi et al., 2020).

Finally, this study found that hypothyroidism triggers testicular dysfunction centrally by affecting the hypothalamic pituitary testicular axis via modulating nesfatin‐1, and downstream MAPK/ERK signaling pathway, and locally by affecting testicular metabolic and redox states, while Se prevents this testicular dysfunction centrally by alleviating hypothyroidism and peripherally by acting directly on testicular tissue.

## 5 CONCLUSION

The hypogonadal state associated with hypothyroidism arises from both central disturbances of the HPT axis and the direct impact of hypothyroidism on testicular tissue. Se had a positive impact on the HPT axis only when it was co‐administered with PTU by reversing the hypothyroid state which further confirms the central impact of hypothyroidism on the HPT. It was evident that the nesfatin‐1 level is disrupted in instances of hypogonadism triggered by hypothyroidism, and this was counteracted, with nesfatin‐1 levels increasing upon hypothyroidism alleviation by Se. As a result, nesfatin‐1 could serve as a potential link between the disrupted HPT axis and hypothyroidism, potentially by influencing the pMAPK/ERK signaling pathway.

### 5.1 Recommendation

Further investigation is required to elucidate the underlying mechanism of how hypothyroidism leads to reduced nesfatin‐1 levels. Also, combining PTU, Se, and a miRNA targeting NUCB2 could ascertain the precise role of nesfatin‐1 as a mediator of selenium's protection against hypothyroid‐induced hypogonadism.

## AUTHOR CONTRIBUTIONS

Rehab Ahmed Ahmed El‐Shaer, Sarah Ibrahim, and Marwa Mahmoud Awad conceived and designed research. Rehab Ahmed Ahmed El‐Shaer, Sarah Ibrahim, Marwa Mohamed Atef, Omnia Safwat El‐Deeb, Yasser Mostafa Hafez, Rania Saed Amer, Passant Medhat Hewady, Norhan Ahmed AbuoHashish, Jehan Abd El‐Hameed El‐Sharnoby, and Marwa Mahmoud Awad performed experiments. Rehab Ahmed Ahmed El‐Shaer, Sarah Ibrahim, Marwa Mohamed Atef, Omnia Safwat El‐Deeb, Yasser Mostafa Hafez, Norhan Ahmed AbuoHashish, Jehan Abd El‐Hameed El‐Sharnoby, and Marwa Mahmoud Awad analyzed data. Rehab Ahmed Ahmed El‐Shaer, Sarah Ibrahim, Rania Saed Amer, Marwa Mohamed Atef, Omnia Safwat El‐Deeb, Passant Medhat Hewady, Jehan Abd El‐Hameed El‐Sharnoby, and Marwa Mahmoud Awad interpreted results of experiments. Rehab Ahmed Ahmed El‐Shaer, Sarah Ibrahim, Norhan Ahmed AbuoHashish, Yasser Mostafa Hafez, and Marwa Mahmoud Awad prepared figures.Rehab Ahmed Ahmed El‐Shaer, Sarah Ibrahim, Marwa Mohamed Atef, Norhan Ahmed AbuoHashish, and Passant Medhat Hewady drafted manuscript. Marwa Mahmoud Awad, and Rehab Ahmed Ahmed El‐Shaer edited and revised manuscript. Rehab Ahmed Ahmed El‐Shaer, Sarah Ibrahim, Marwa Mohamed Atef, Omnia Safwat El‐Deeb, Yasser Mostafa Hafez, Rania Saed Amer, Passant Medhat Hewady, Norhan Ahmed AbuoHashish, and Marwa Mahmoud Awad approved final version of manuscript.

## FUNDING INFORMATION

For this study, the authors received no specific funds from financial sources.

## CONFLICT OF INTEREST STATEMENT

The authors did not disclose any potential conflicts of interest.

## Data Availability

On request, the corresponding author will provide the data that backs up the study's findings.
